# DNA methylation: The epigenetic mechanism of Alzheimer's disease

**DOI:** 10.1002/ibra.12121

**Published:** 2023-08-10

**Authors:** Hao‐Yue Qin, Jiao‐Yan Liu, Chang‐Le Fang, Yan‐Ping Deng, Ying Zhang

**Affiliations:** ^1^ Department of Anesthesiology Southwest Medical University Luzhou Sichuan China; ^2^ Faculty of Health Sciences University of Adelaide Melbourne VIC Australia; ^3^ State Key Laboratories for Quality Research in Chinese Medicines, Faculty of Pharmacy Macau University of Science and Technology Macau China

**Keywords:** Alzheimer's disease, DNA methylation, epigenetics

## Abstract

Nowadays, with the development of the social health care system, there is an increasing trend towards an aging society. The incidence of Alzheimer's disease (AD) is also on the rise. AD is a kind of neurodegenerative disease that can be found in any age group. For years, scientists have been committing to discovering the cause of AD. DNA methylation is one of the most common epigenetic mechanisms in mammals and plays a vital role in the pathogenesis of several diseases, including tumors. Studying chemical changes in the epigenome, or DNA methylation can help us understand the effects of our environment and life on diseases, such as smoking, depression, and menopause, which may affect people's chances of developing Alzheimer's or other diseases. Recent studies have identified some crucial genes like *ANK1*, *RHBDF2*, *ABCA7*, and *BIN1*, linking DNA methylation to AD. This review focuses on elucidating the relationship between DNA methylation and the pathogenesis of AD and provides an outlook on possible targeted therapeutic modalities.

## Graphical abstract

This study discusses possible pathways that may affect the brain after methylation of gene loci highly associated with Alzheimer's disease (AD). Many literature studies have shown that estrogen and aging can lead to DNA methylation, including key genes such as *APP* and *APOE*. Through these, we found that DNA methylation can be used as an early screening method for AD.

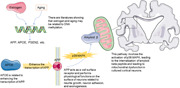

## 1 INTRODUCTION

Alzheimer's disease (AD) is a neurodegenerative disease in which patients usually show a progressive loss of situational memory and cognitive function, which subsequently leads to deficits in verbal and visuospatial skills, often accompanied by behavioral disturbances such as apathy, aggression, and depression.^1^ According to the statistics, ~50 million people worldwide suffer from AD and nearly 10 million new cases occur each year.^2^ Many people may regard AD as a disease that can only be found at higher ages (medically known as late‐onset AD, LOAD), but the cruel truth is that there are still many young people sadly diagnosed with AD (medically known as early‐onset AD, EOAD).^3^, ^4^ What's worse, many studies have revealed that people with EOAD may suffer from more severe‐neuropsychiatric symptoms than those with LOAD.^3^, ^5^ At present, the treatment of AD is mainly through drugs or cholinesterase inhibitors to improve cognitive dysfunction.^6^ Although this method has been proven to be effective, it cannot radically cure AD from its pathogenesis.^7^ Therefore, the scheme of treating AD from the pathogenesis is still worthy of further exploration.

DNA methylation is the addition of a methyl group to cytosine next to guanine, a form of epigenetic regulation that regulates the genes used by the cell's transcription machinery and their expression levels.^8^ Studies have shown that DNA methylation is an important way for environmental factors to disrupt neurodegenerative genes, which can greatly increase the risk of neurodegenerative diseases.^9^ In addition, the degree of DNA methylation differs between the normal population and those with AD.^10^ These studies have shown that there is a relationship between DNA methylation and the pathogenesis of AD. Thus, the aim of this review is to explore the possible link between DNA methylation and the onset of AD as well as to offer a new possibility for the treatment of AD.

## 2 HYPOTHESIS OF AD PATHOGENESIS

AD is a disease caused by several risk factors such as increasing age, genetic factors, cerebrovascular diseases, diabetes, hypertension, obesity, and dyslipidemia.^1^, ^11^, ^12^, ^13^ It is characterized by neuritic plaques and neurofibrillary tangles.^14^ Uncovering what causes these plaques and tangles to form and what role they play in disease progression is currently a research hotspot in this field, and it is essential for the development of prevention and treatment strategies. Various mechanisms of hypoxia and oxidative stress are currently the focus of research on the pathogenesis of AD, such as those caused by vascular and mitochondrial lesions, but their specific mechanisms lack linkage to other mechanisms.^15^ Furthermore, AD is a disease that is strongly linked to genetics. According to one study, healthy older adults with a family history of AD had more subjective memory impairment and were thus at a higher risk of developing the disease.^16^ This effect is able to be detected by imaging and molecular biology tests.^17^, ^18^

The current hypothesis includes the amyloid cascade hypothesis, tau hypothesis, and cholinergic hypothesis, among others.^12^, ^19^, ^20^, ^21^ The amyloid cascade hypothesis is one of the most widely accepted mechanisms of AD pathogenesis and is centered on the imbalance of amyloid β (Aβ) peptide production and clearance, leading to the neurodegenerative pathology of AD.^1^ Aβ peptide is a series of peptide proteins formed by cleavage of amyloid precursor protein (*APP*) by β‐ and γ‐secretase, which is a memory enhancer at physiological concentrations but causes memory impairment, oxidative damage, blood–brain barrier damage, neurogenic fiber tangles, and amyloid plaque formation when concentrations exceed normal concentrations.^22^

Related genetic studies have shown that mutations in dominant genes such as *APP*, presenilin 1 (*PSEN1*), and presenilin 2 (*PSEN2*) located on autosomes are closely related to the imbalance process of Aβ peptide.^21^ Methylation of genes such as *APP* and *PSEN2* has also been found to be strongly associated with the development of AD.^23^, ^24^ The hypothesis is excellent in suggesting an important role for DNA methylation in the pathogenesis of AD.

## 3 DNA METHYLATION

DNA methylation is a major epigenetic mechanism that normally represses gene expression, whereas demethylation induces gene reactivation and expression, thereby regulating gene expression. Many studies have identified that DNA methylation can control gene expression by causing changes in chromatin structure, DNA conformation, DNA stability, and the way DNA interacts with proteins without altering the gene sequence. DNA methylation has been used for early screening of certain cancers and tumors, and screening for genetic diseases such as Prader–Willi syndrome.^25^, ^26^, ^27^ The current detection methods include methylation‐specific polymerase chain reaction (PCR), bisulfite‐sequencing PCR, and high‐resolution melting.

DNA methylation occurs widely in cytosine‐guanine dinucleotide separated by phosphate (CpG)‐rich sequences, especially in satellite DNAs, repetitive elements, gene bodies, and nonrepetitive intergenic sequences where CpG sites are highly methylated.^28^ CpG island (CGI) is a segment of DNA sequence rich in CpG dinucleotides, which usually serves as the promoter of a gene and contains almost all housekeeping genes, as well as a portion of tissue‐specific and developmental regulatory genes.^29^ The methylation status of the CGI located at the transcription start site (TSS) determines the expression status of the gene. In general, genes that are blocked have highly methylated CGI around their TSS, whereas transcribed genes have hypomethylated CGI around their TSS, even if the CGI within the gene is hypermethylated.^28^ The altered methylation status of CGI provides an important safeguard mechanism for selective gene expression, as well as temporal and spatial specific expression. Although the CGI around the TSS is enriched in CpG sites, the CpG dinucleotides in them are rarely DNA methylated, which may be related to their unique transcriptional initiation and specific chromatin configuration. For example, among the relevant regulatory proteins that modify the chromatin morphology of CGI are included proteins that specifically bind nonmethylated CpG.^30^ Furthermore, aberrant hypermethylation of CGI is often associated with silencing of tumor suppressor genes in cancer.^31^ In addition to determining gene expression, DNA methylation also plays an important role in the formation of higher chromatin structures, such as the specific recognition of methylated DNA sequences by various methyl‐CpG‐binding proteins and the recruitment of histone modification complexes, thereby stabilizing gene expression patterns and maintaining genomic integrity.^28^ In addition to CpG methylation, non‐CpG methylation also has important effects on the human body, especially the brain.^32^ Non‐CpG methylation accounts for about 35% of the total methylation in the adult brain and can even reach 50% of the total methylation in neuronal tissue. Non‐CpG methylation in neurons is aligned and accumulated during the neurodevelopment of the human brain until aging, promoting the role of de novo DNA methyltransferases (*DNMTs*), and maintaining the normal function of the adult brain.^33^, ^34^

Mammalian DNA methylation is mediated by *DNMT1*, *DNMT2*, and *DNMT3B*, all of which contain multiple structural domains that are divided into two main parts according to function, the C‐terminal domain that mediates catalysis, and the N‐terminal domain that mediates targeting and activity regulation.^28^, ^35^ Upon nonspecific binding of *DNMTs* to DNA, the C‐terminal structural domain specifically recognizes the DNA sequence through the targeting structural domain therein and subsequently catalyzes the binding of the recruited methyl donor *S*‐adenosylmethionine (*SAM*) to the fifth carbon of the flipped cytosine base, thereby mediating methyl transfer to form 5‐methylcytosine.^35^ *DNMT3A* and *DNMT3B* primarily target previously un cytosines of previously unmethylated CpG dinucleotides for de novo methylation, while they also have good binding properties for hemimethylated DNA. *DNMT1*, on the other hand, is mainly stable at high concentrations in dividing cells and maintains the methylation state of the corresponding site by targeting bound hemimethylated DNA.^36^ The N‐terminal structural domain is significantly different in the *DNMT1* and DNMT3 families compared with the C‐terminal structural domain.^37^ As the C‐terminal domain of mammalian *DNMT* does not recognize target DNA regions with strong sequence specificity, the targeting and activity regulation functions of the N‐terminal domain play a central role in the methylation process.^38^ The N‐terminal domain is primarily involved in the nuclear localization of enzymes; mediates interactions with other proteins, with regulatory nucleic acids (e.g. noncoding RNAs) and with chromatin modifications; performs posttranslational modifications; and it is involved in the regulation of enzyme activity and specific conformation.^35^, ^37^

## 4 POSSIBLE PATHWAYS OF DNA METHYLATION LEADING TO THE PATHOGENESIS OF AD

Abnormal DNA methylation can lead to age‐related diseases such as AD. On the one hand, the related genes that lead to the onset of AD are hypomethylated with increased expression of β‐*APP* (dl‐app) lyase (*BACE*). Meanwhile, genes that inhibit the onset of AD, such as nuclear export protein (*NEP*) and ankyrin 1 (*ANK1*), are highly methylated and reduced in expression, resulting in the overproduction and deposition of β‐amyloid.^39^ *SAM* is the main methyl donor in vivo. *SAM*/homocysteine circulation disorder often leads to abnormal DNA methylation and promotes the occurrence of AD. Oxidative damage to DNA is extremely detrimental to the function and survival of neurons and DNA methylation abnormalities exacerbate oxidative damage to DNA and prevent DNA repair. It has been shown that elevated DNA methylation associated with the neuropathology of AD spans the *HOXA* gene cluster on chromosome 7.^40^ In addition, studies have found that changes in DNA methylation in the superior temporal gyrus of patients with AD highlight the new sites and mechanisms that characterize the pathogenesis of AD.^41^ These facts highlight the role of epigenetic variation in AD.^40^ A growing body of research suggests that DNA methylation of specific genes is strongly associated with the development of AD and these genes are mainly associated with aging and sex.

### 4.1 DNA methylation and aging‐related genes in AD

AD, as a neurodegenerative disease, has been discussed in studies over the years, linking its onset to age. There are studies showing that ~2% of CpH sites undergo DNA methylation changes associated with aging, suggesting that ~0.5% of CpG sites undergo significant changes with aging.^42^ Although only a small fraction of the genome undergoes methylation changes in cytosines in relation to age, quantitative changes can lead to qualitative changes.^42^ Among them, DNA methylation of *APP*, Apolipoprotein E (*APOE*), *PSEN2*, and other aging‐related genes are closely related to the occurrence of AD.

**Figure 1:**
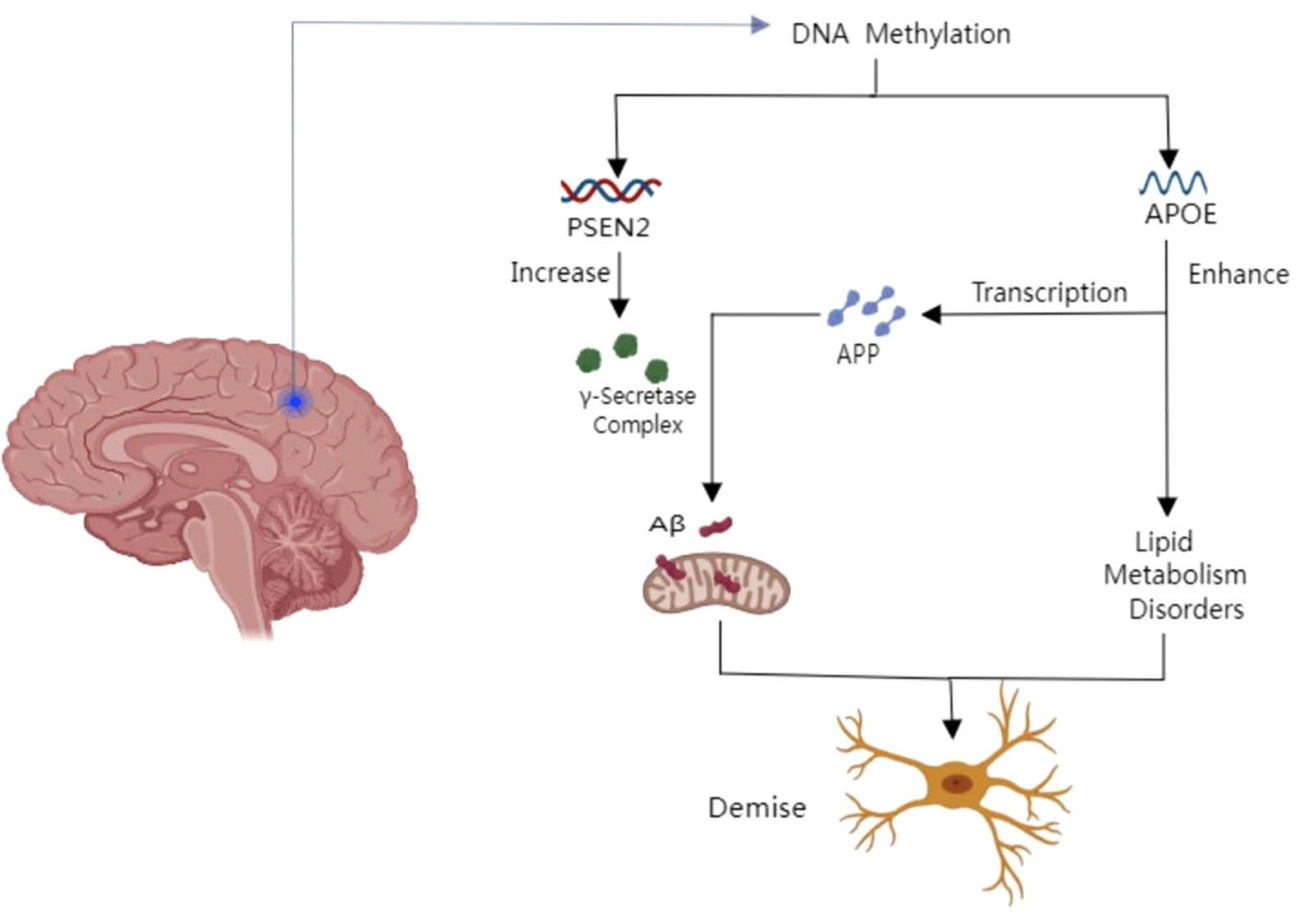
Mechanism of DNA methylation in aging‐related genes and Alzheimer's disease. *APP*, amyloid precursor protein; *APOE*, apolipoprotein E; Aβ, amyloid β peptide; *PSEN2*, presenilin 2. [Color figure can be viewed at wileyonlinelibrary.com]

*APP* acts as a cell surface receptor and performs physiological functions related to neurite growth, neurite adhesion, and axon formation on the surface of neurons. Interactions between *APP* molecules on adjacent cells promote synapse formation. It is involved in cell motility and transcriptional regulation through protein–protein interactions. This pathway involves the activation of p38 mitogen‐activated protein kinase, leading to the internalization of Aβ peptide and leading to mitochondrial dysfunction in cultured cortical neurons.^43^ *APOE* is an apolipoprotein, a protein related to lipid particles, which plays a role in lipoprotein‐mediated lipid transport between organs mainly through plasma and interstitial fluid. It is the core component of plasma lipoproteins and participates in their production, transformation, and clearance.^44^ The development of neurodegenerative diseases has an effect on lipid metabolism disorders in the brain.^45^, ^46^ Studies have found that a reduction in *APOE* methylation following AD brain death may suggest that abnormal epigenetic changes in *APOE* are associated with the risk of AD.^44^, ^47^, ^48^ Meanwhile, *APOE* has been associated with enhanced transcription of *APP*. *PSEN2* is currently speculated that it may be the catalytic subunit of the γ‐secretase complex. This is an endonuclease complex and it can catalyze intramembrane cleavage of integral membrane proteins such as the Notch receptor and *APP*. The γ‐secretase complex may play a role in intracellular signal transduction and gene expression or in the linkage of chromatin to the nuclear membrane. It may also play a role in the cytoplasmic distribution of proteins. The whole protein of the γ‐secretase complex plays a role in acting as a calcium leak channel, thus allowing the passive movement of calcium from the endoplasmic reticulum to the cytoplasm and participating in calcium homeostasis. It is a regulator of mitochondrial‐endoplasmic reticulum membrane binding. It also regulates the shuttling of calcium ions between the endoplasmic reticulum and the mitochondria. DNA methylation at multiple AD loci in the brain has been pathologically linked to AD. These results provide further evidence that disruption of DNA methylation is involved in the pathological process of AD.^49^ A diagram of the mechanism is shown in Figure 1.

### 4.2 DNA methylation in sex‐related genes and AD

One study showed that two‐thirds of Alzheimer's patients are women and that this difference is mainly related to estrogen.^50^ Literature has shown that estrogen can affect learning, memory, and mood.^51^ In addition, it has been shown that neuronal production of 17β‐estradiol achieves modulatory effects on synaptic and spine density, excitatory synaptic transmission, and long‐term dementia, as well as modulation of hippocampal‐dependent recognition memory, spatial reference memory, and contextual fear memory through estrogen receptor‐α (ERα)‐mediated fast kinase signaling and CREB‐BDNF signaling pathways.^52^, ^53^ ERα and ERβ are expressed in a variety of brain regions and ERα undergoes developmental and specific expression changes in brain regions.^54^, ^55^ Chinese researchers have found that methylation of the ERα promoter can inhibit the expression and transcription of ERα messenger RNA, which is related to the impairment of cognitive function and quality of life in AD patients.^54^ Several researchers have identified DNA methylation‐related silencing of a number of target genes and these hypermethylated targets suggest that the cyclic AMP response element binding protein (*CREB*) activation pathway and axon initiation fragments may contribute to AD.^56^ Thereby, we hypothesize that the promotion of DNA methylation may be related to estrogen. There are supportable studies showing that over 95% of age‐related DNA methylation changes in the hippocampus are gender specific, which suggests that methylation would show a more severe age predisposition in women, coinciding with the greater susceptibility of women to develop AD.^42^, ^57^

### 4.3 DNA methylation and cytokine regulation in AD

The role of inflammatory mechanisms triggered by cytokines in the pathogenesis of AD has been somewhat elucidated.^58^

Tumor necrosis factor‐α (*TNF‐α*) plays a crucial role in promoting inflammation and pro‐apoptosis. *TNF‐α* induces neuronal apoptosis through *TNF‐α* p55 receptors constitutively expressed in a pan‐neuronal pattern and inflammation through *TNF‐α* p75 receptors.^59^ Therefore, sustained activation of *TNF‐α* p55 or p75 receptors can have deleterious consequences on the brain. Some experimental data suggest that lower methylation levels and less restriction of the *TNF‐α* promoter in the brains of AD patients lead to elevated levels of *TNF‐α* expression, which may contribute to the pathogenesis of AD.^60^, ^61^

Elevated levels of interleukin 6 (*IL‐6*), a cytokine with pro‐ and anti‐inflammatory functions, lead to cognitive decline and AD.^62^ The formation of β‐amyloid plaques and tau proteins in AD induces neuronal cells to secrete immune factors such as *IL‐6,* while increased levels of immune factors such as *IL‐6* promote the formation of β‐amyloid plaques and phosphorylated tau proteins.^63^ It has been shown that low DNA methylation levels within the *IL‐6* promoter significantly affect the expression of IL‐6.^64^ The results not only provide a basis for the close association of DNA methylation of cytokines with the pathogenesis of AD but also further expand the understanding of the amyloid cascade hypothesis and tau hypothesis on the pathogenesis of AD.

### 4.4 DNA methylation in mitochondria and AD

The role of oxidative stress and mitochondrial dysfunction in the pathological process of neurodegenerative diseases has received much attention in recent years.^65^ It has been shown that, in addition to the nuclear DNA, mitochondrial DNA (mtDNA) methylation also has an important impact on the course of AD.^66^ We will now focus on the role of mtDNA methylation in the development of AD.

Compared with nuclear genes, mtDNA is in turn more susceptible to oxidative stress damage due to the lack of histone protection and complex DNA repair mechanisms. The accumulation of reactive oxygen species (ROS) due to dysfunctional mitochondrial electron transport is the primary factor of oxidative damage. On the one hand, when mtDNA is exposed to oxidative stress, it activates the transcription factors NRF1 and PGC1‐α, which promote the expression of the mitochondrial methyltransferase DNMT1, which in turn hypermethylates genes in mtDNA, mainly those encoding cytochrome B (*CytB*), cyclooxygenase‐2 (*COX‐2*), and 12S ribosomal RNA (rRNA) in AD patients, thereby suppressing gene expression.^67^, ^68^ *MT‐CYTB* and *MT‐COX II* genes are important genes encoding respiratory chain Complex III and Complex IV, respectively, whereas the mitochondrial 12S rRNA genes encode proteins that play an important role in the transcription and translation process of mitochondrial protein synthesis. When these genes are overmethylated, mitochondrial respiratory chain protease synthesis is blocked, resulting in impaired electron transfer and ROS accumulation, which in turn increases oxidative damage to mtDNA, creating a vicious cycle. On the other hand, the accumulation of Aβ42 in the mitochondria of AD patients decreases the permeability of the mitochondrial membrane and reduces the production of ATP, which in turn blocks the electron transport process and leads to the accumulation of ROS.^66^ In addition, the accumulation of Aβ interferes with the Krebs cycle by inhibiting the activity of 2‐oxoglutarate dehydrogenase, which in turn activates DNA demethylases of the 2‐oxoglutarate‐dependent oxygenase family, and the intermediate metabolites succinate and fumarate in the Krebs cycle, which in turn inhibit the activity of 2‐oxoglutarate‐dependent oxygenases.^69^ It can be hypothesized that there is an association between impairment of mitochondrial energy metabolism and disturbances in the regulatory mechanisms of methylation and demethylation of mtDNA.

## 5 DNA TARGETED THERAPY

Targeted therapies for DNA methylation are expected to be used in the treatment of tumors. Although there are over 14,000 publications on the association of biomarkers of DNA methylation with cancer, very few of them have been applied in clinical testing.^70^ Clinical treatments for DNA demethylation in diseases such as tumors usually use chemotherapy that has the effect of reducing overall DNA methylation levels within cells. Due to the indiscriminate reduction of DNA methylation levels, it is difficult to control the therapeutic effects and side effects of such treatments. For example, myelodysplastic syndrome treatment with demethylating drugs such as azacitidine and decitabine often results in complications such as myelosuppression and infection.^71^, ^72^ Therefore, DNA demethylation requires targeted regimens to enable more precise efficacy. There are no reports of demethylation therapy for AD. This idea is presented in this study but the actual applicability of this therapy to AD still requires experimental confirmation.

**Figure 2:**
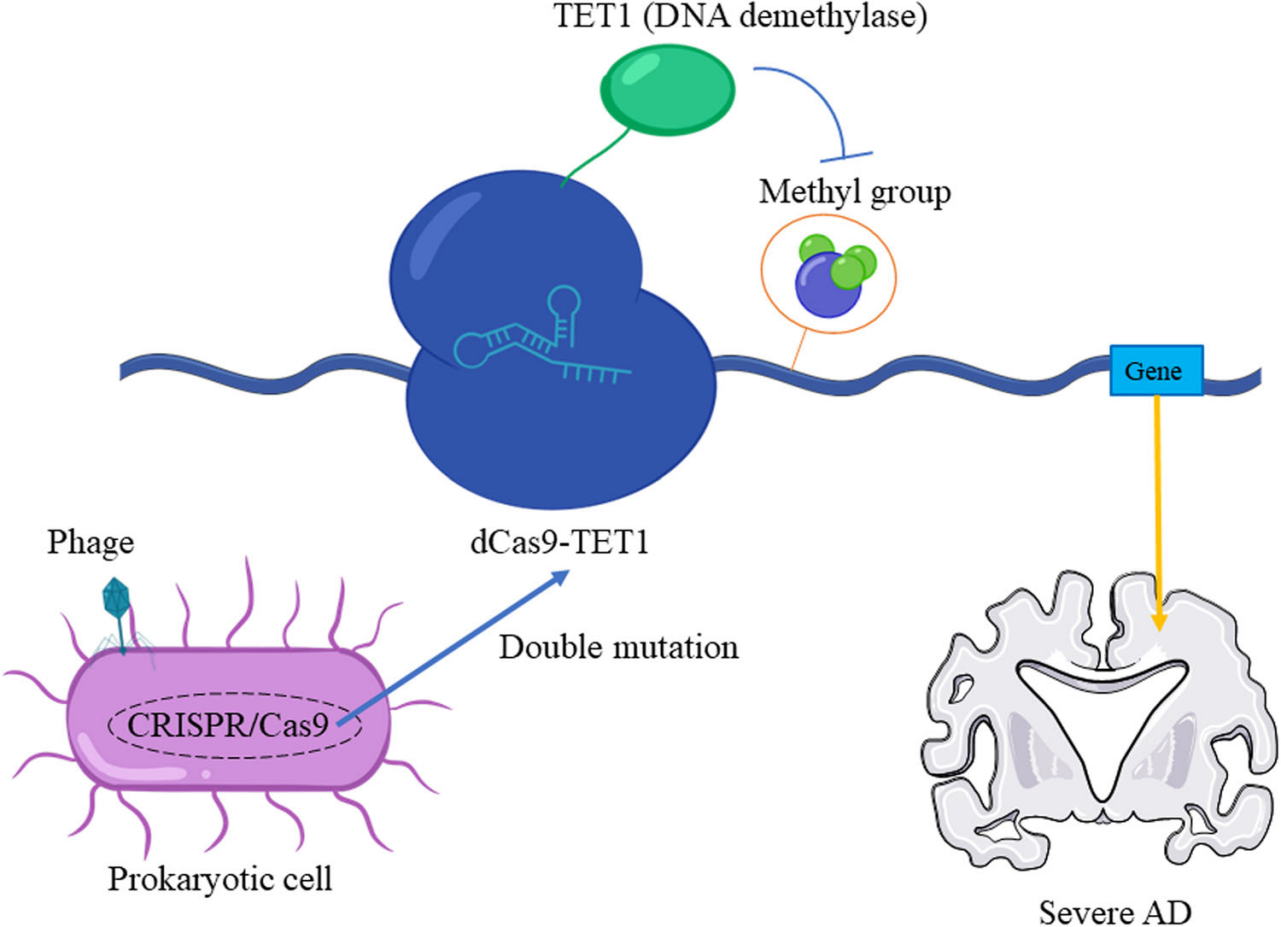
Targeted therapies for DNA methylation (demethylation of *PSEN2* as an example). dCas9‐TET1 is a DNA demethylation modification system that enables rapid promoter demethylation. dCas9‐TET1 demethylation modification system drives transcriptional activation of target genes in various cell types including embryonic stem cells, cancer cell lines, fibroblasts, and primary neurons via TET1 (DNA demethylation motif). AD, Alzheimer's disease; CRISPR/Cas9, clustered regularly interspaced short palindromic repeats (CRISPR)/CRISPR‐associated protein 9; *PSEN2*, presenilin 2; TET1, ten‐eleven translocation 1. [Color figure can be viewed at wileyonlinelibrary.com]

The clustered regularly interspaced short palindromic repeats (CRISPR)/CRISPR‐associated (Cas) protein (CRISPR/Cas) system is a natural immune system for prokaryotes. When invaded by a virus, some bacteria are able to store a small segment of the virus's gene in a storage space called CRISPR in their own DNA. When a virus invades again, the bacterium is able to recognize the virus based on the stored fragment and disable it by cutting off the virus’ DNA.^73^ In this case, dCas9 is the result of a double mutation in the nuclease Cas9 that produces blunting and death. dCas9‐ten‐eleven translocation 1 (*TET1*) is one of the new technologies for editing the epigenome, a system consisting of the catalytic structural domain (CD) of the demethylase *TET1* fused to dCas9. dCas9‐*TET1* allows rapid demethylation and induces upregulation of target gene expression.^74^, ^75^, ^76^ Thus, the properties of dCas9‐*TET1* can be used as a tool for targeted demethylation therapy for AD. Here is an example of demethylation of *PSEN2*, a mutation in *PSEN2* that results in the production of large amounts of β‐amyloid fragments. These β‐amyloid fragments adhere to each other and are deposited in the brain to form amyloid plaques and cause the tau protein to malfunction. The tau protein particles then adhere to each other, forming neuronal fibrillary tangles that eventually lead to brain cell death and display the symptoms of AD. In contrast, demethylation of *PSEN2* is effective in reducing the production of β‐amyloid and thus relieving symptoms (Figure 2). However, this therapy is not currently used in the treatment of AD and there is no literature on the subject.

## 6 CONCLUSIONS AND PROSPECT

This study summarizes the main causes of AD: extraneuronal toxic amyloid deposits, interneuronal neurogenic fiber tangles composed of hyperphosphorylated tau, region‐specific reduction in brain glucose metabolism, synaptic dysfunction, and mitochondrial dysfunction.^77^ At the same time, we describe the role that DNA methylation plays in these causes. After decades of research, it has been found that the pathogenesis of AD is closely related to Aβ deposition, tau tangles, neuroinflammatory mechanism theory, neurovascular damage theory, oxidative stress, and so on.

DNA methylation plays an important regulatory role in cell differentiation, maturation, and aging. Numerous studies have shown that DNA methylation plays a role in the development and progression of AD.

Despite revealing the relevance of DNA methylation in cytokine regulation in the human brain, the current findings also raise new questions that deserve further investigation. One of these focuses on figuring out which cellular DNA methylation can lead to AD since in the cortex the number of microglia is too low.^78^ Some literature suggests that such cells may be *CD4*+ lymphocytes present in the cortex. However, there is no strong evidence in this literature to suggest a pathological link between *CD4*+ lymphocytes and AD.^79^, ^80^

Many experimental results suggest that the study of epigenetic signatures can help characterize novel genomic regions associated with disease. Future challenges in this field include the discovery of effective strategies and the integration of epigenetic and transcriptomic features with genetic data sets. This will allow a better understanding of the role of different forms of variation in AD.^41^ We, therefore, believe that DNA methylation holds great promise for elucidating the pathogenesis of AD as well as its treatment. In addition, DNA methylation has shown promising clinical applications in the diagnosis, as well as the treatment of AD. On the one hand, we can analyze 5′methylcytosine and histone modification events at the genome‐wide level using DNA methylation sequencing for early screening of AD.^81^, ^82^ On the other hand, we can achieve treatment of AD by inhibiting DNA methylation. Studies have shown that the use of *DNMT* inhibitors has a role in the treatment of cancer.^83^ Thus, we speculate that AD can also be treated by inhibitors of *DNMTs*, *DNMT1* inhibitors, especially. There is no literature on demethylation therapy for AD, so we hope that this literature will provide ideas for more researchers.

## AUTHOR CONTRIBUTIONS

Hao‐Yue Qin analyzed most of the data and wrote the initial draft of the study. Jiao‐Yan Liu contributed to the revision. Chang‐Le Fang and Yan‐Ping Deng contributed the central idea. Ying Zhang provided corrections and guidance for this article.

## CONFLICT OF INTEREST STATEMENT

The authors declare no conflict of interest.

## ETHICS STATEMENT

Not applicable.

## Data Availability

Data sharing is not applicable to this article, as no new data were created or analyzed in this review.
